# The impact of CT-based adipose tissue distribution and sarcopenia on treatment outcomes in patients with high-risk soft tissue sarcoma

**DOI:** 10.1186/s12885-025-14050-x

**Published:** 2025-04-11

**Authors:** Luc M. Berclaz, Dorit Di Gioia, Michael Völkl, Vindi Jurinovic, Alexander Klein, Hans Roland Dürr, Thomas Knösel, Bianca Teodorescu, Stefan Enßle, Michaela Rippl, Michael von Bergwelt-Baildon, Wolfgang G. Kunz, Lars H. Lindner, Anton Burkhard-Meier

**Affiliations:** 1 Department of Internal Medicine III, University Hospital, LMU Munich, Munich, Germany; 2 German Cancer Consortium (DKTK), Partner Site Munich, Munich, Germany; 3 Institute for Medical Information Processing, Biometry, and Epidemiology, University Hospital, LMU Munich, Munich, Germany; 4 Orthopaedic Oncology, Department of Orthopedics and Trauma Surgery, University Hospital, LMU Munich, Munich, Germany; 5 Institute of Pathology, LMU Munich, Munich, Germany; 6 Department of Internal Medicine II, University Hospital, LMU Munich, Munich, Germany; 7 Department of Internal Medicine IV, University Hospital, LMU Munich, Munich, Germany; 8 Department of Radiology, University Hospital, LMU Munich, Munich, Germany; 9 Bavarian Cancer Research Center (BZKF), Munich, Germany

**Keywords:** RECIST, EORTC-STBSG, Chemotherapy, Regional hyperthermia, Soft tissue sarcoma, Skeletal muscle index, Total fat index, Fat to muscle ratio

## Abstract

**Background:**

The prognostic and predictive value of obesity and sarcopenia remains poorly defined in patients with high-risk soft tissue sarcoma (HR-STS). We sought to correlate clinical outcomes with CT-based body composition parameters in patients with HR-STS undergoing a multimodal preoperative therapy. The impact of radiologic and histopathologic response to preoperative treatment was correlated with individual fat and muscle distribution.

**Methods:**

Patients with locally advanced non-abdominal HR-STS and treatment with preoperative chemotherapy + regional hyperthermia (RHT) +/- radiotherapy (RT) followed by surgery between 2015 and 2022 were retrospectively evaluated. Body composition parameters measured on baseline CT scans were correlated with clinical outcomes including event-free survival (EFS) and overall survival (OS) as well as radiologic and histopathologic treatment response.

**Results:**

A total of 85 patients were included. Body composition parameters showed no significant correlation with radiologic or histopathologic treatment response. High total fat indices such as the total fat index (TFI, HR 3.56, *p* = 0.005) and high total fat to muscle ratio (FMR, HR 3.22, *p* = 0.020) were strongly associated with poor OS. Parameters for sarcopenia including skeletal muscle index (SMI) were not significantly linked to survival outcomes.

**Conclusion:**

High fat indices and a high FMR are strong predictors of poor OS in patients with HR-STS. Larger studies are warranted to further clarify the prognostic impact of sarcopenia and the predictive value of body composition parameters on preoperative treatment response.

**Supplementary Information:**

The online version contains supplementary material available at 10.1186/s12885-025-14050-x.

## Introduction

Soft tissue sarcomas (STS) are rare tumors with multiple distinct histological subtypes. They account for approximately 1% of adult malignancies [1]. Despite optimal local treatment, almost half of patients with high risk features (HR-STS: Tumor diameter 5 cm or larger, grade 2 or 3 according to Fédération Nationale des Centres de Lutte Contre le Cancer (FNCLCC), deep to the fascia) will die within five years of their diagnosis [2, 3]. Perioperative chemotherapy is often recommended in addition to surgery and radiotherapy in patients with HR-STS [4, 5]. The addition of regional hyperthermia (RHT) to neoadjuvant chemotherapy has shown to improve both response and survival in HR-STS and is therefore being considered as an additional treatment option [6, 7]. Moreover, preoperative radiotherapy is widely accepted as standard treatment for patients with extremity sarcomas undergoing limb-sparing surgery [8].

Predictive factors associated with response to systemic treatment and outcomes are currently limited and mostly based on clinical parameters such as age, grade, performance status and histology [2, 9]. Several studies have analyzed the role of the body composition regarding response to systemic treatment and outcomes in cancer patients, with varying results so far. Sarcopenia is defined as a loss of skeletal muscle mass and function and correlates with a low skeletal muscle index (SMI) and low muscle radiation attenuation (MRA), defined as low muscle density measured in Hounsfield units on computed tomography (CT) scans [10]. Sarcopenia was associated with adverse outcomes across multiple cancer types including advanced and metastatic STS undergoing palliative systemic therapy [11–14]. Moreover, sarcopenia resulted in higher chemotherapy-related toxicities and impaired health-related quality of life [15, 16].

In contrast, the evidence is less conclusive on the role of obesity in the response to systemic therapy and treatment outcomes in STS. Obesity is an established risk factor for a wide variety of malignancies and associated with a proinflammatory and prothrombotic systemic milieu [17, 18]. In contrast to subcutaneous fat (SF), visceral fat (VF) is linked to insulin resistance, metabolic syndrome, and increased risk of cancer mortality [17, 19]. Although not optimally reflective of the body composition and different fat compartments, high body mass index (BMI) has also been linked to an increased incidence, worse overall survival (OS), and increased postoperative complications across multiple cancer types [20]. On the other hand, several studies suggest a protective effect of VF due to an increased sensitivity to systemic therapy such as immune checkpoint inhibitors, also described as the obesity paradox [21]. A recently published meta-analysis failed to demonstrate an association of obesity and oncologic outcomes, which underlines the inconclusive data regarding the relationship between nutritional status and treatment response or outcomes in STS [13]. Optimal parameters and cut-offs for obesity are currently missing.

Aim of this study was to analyze and correlate CT-based specific body composition parameters with treatment outcomes in a large and well-characterized cohort of patients with HR-STS undergoing a multimodal treatment approach. In addition, we sought to correlate the role of the body composition with radiologic and histopathologic treatment response according to the RECIST and EORTC-STBSG scoring systems [22, 23].

## Materials and methods

### Patient selection and treatment

**Fig. 1:**
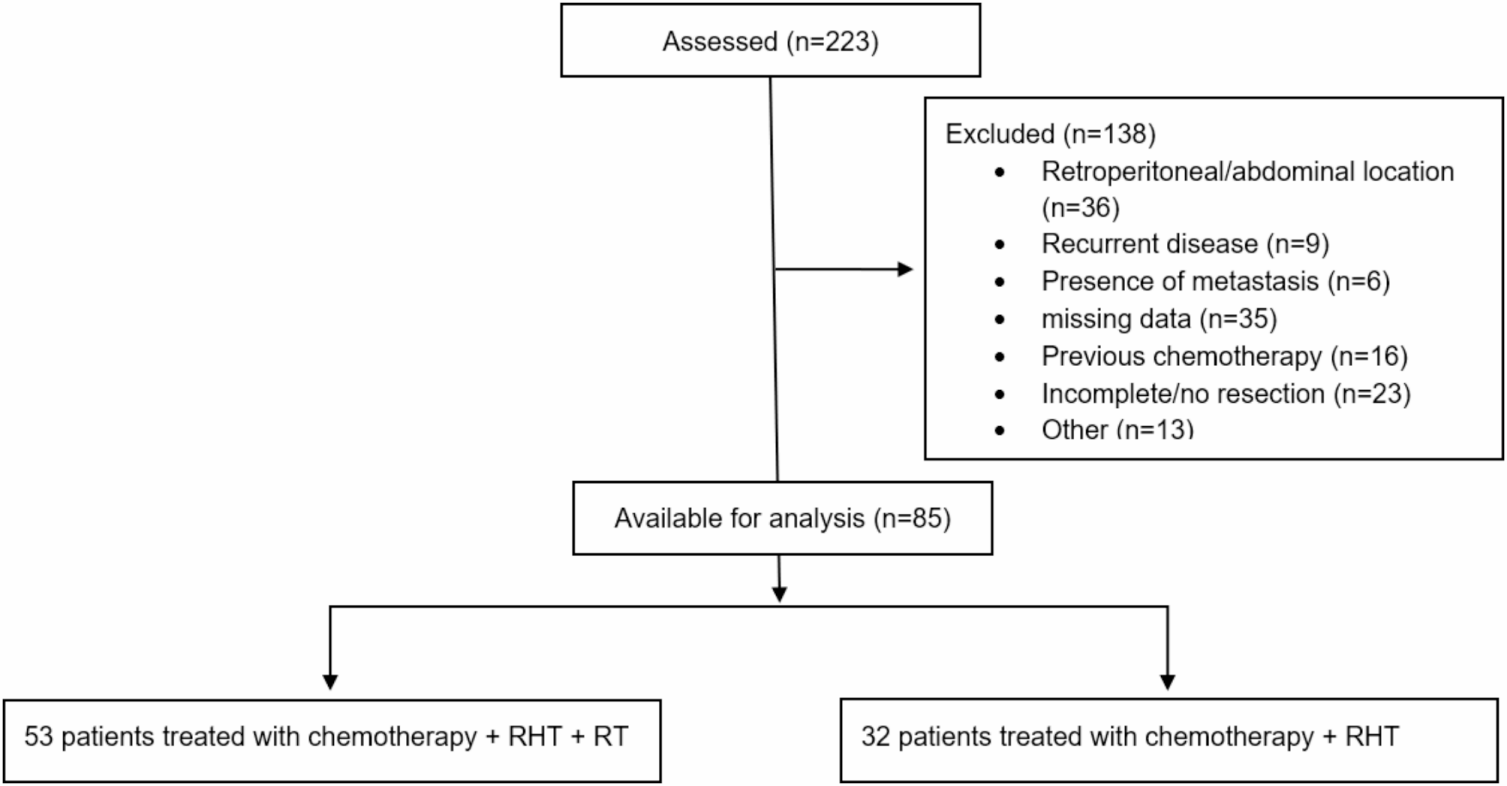
Flowchart of evaluated patients. Other: no RHT (*n* = 2), only 1 cycle of chemotherapy and RHT (*n* = 3), Isolated limb perfusion (*n* = 3), Deviation from the treatment protocol (*n* = 4), previous malignancy (*n* = 1)

An exploratory retrospective cohort study design was chosen to address the research question. Eligible patients had pathologically confirmed locally advanced extremity, trunk or head and neck HR-STS without evidence of metastasis and were treated at our institution between January 2015 and May 2022. Patients with abdominal sarcomas were excluded from this study due to a potential bias in the analysis of baseline CT scans at the level of the third lumbar vertebra (L3). Clinical, pathologic, and outcomes data were extracted from our clinical sarcoma database. Patients received up to eight cycles of either doxorubicin in combination with ifosfamide (AI) or doxorubicin in combination with dacarbazine (AD) for leiomyosarcoma or in case of impaired renal function. Patients < 60 years of age received 60mg/m^2^ of doxorubicin per cycle and 9 g/m^2^ of ifosfamide (decreased to 6 g/m^2^ for cycles 5–8) or 1200mg/m^2^ of dacarbazine (decreased to 900 mg/m2 for cycles 5–8). The standard dose for patients ≥ 60 years was 60mg/m^2^ of doxorubicin combined with either 6 g/m^2^ of ifosfamide or 900 mg/m^2^ of dacarbazine per cycle. All patients were treated with chemotherapy in combination with RHT. RHT aiming for tumor temperatures elevating to 40°-43 °C for 60 min was given twice per chemotherapy cycle. Quality and safety of hyperthermia was ensured by the European Society for Hyperthermic Oncology (ESHO) guidelines [24]. The BSD-2000 hyperthermia system (PYREXAR Medical, Salt Lake City, UT, USA) was used. Surgery was generally performed after four cycles of chemotherapy and RHT. In case of tumor progression detected on CT imaging after two cycles, chemotherapy was discontinued, and surgery was performed earlier. Radiotherapy was used in a pre- or postoperative setting in patients with extremity sarcomas or in selected non-extremity cases to enhance local tumor control after discussion in our multidisciplinary sarcoma tumor board. All exclusion criteria can be seen in Fig. 1.

### Radiological assessments and AI-based body composition analysis

CT staging was performed before start (≤ 3 weeks) and after two cycles of treatment. CT imaging was performed with contrast enhancement. Staging was conducted using a Siemens SOMATOM Drive Dual Source CT scanner, covering the chest, abdomen, and pelvis in the portal-venous contrast phase with CAREkV for automatic tube voltage adaptation. CT reconstructions were performed with a 2 mm slice thickness for lung kernel and 3 mm for soft tissue kernel. The CoreSlicer web-based software package (CoreSlicer, version 1.0, Montreal, Canada) was used on baseline imaging to measure body composition parameters including the volumes of both psoas muscles, bilateral abdominal and paraspinal muscles (total muscle area) and subcutaneous and visceral fat (total fat area). The level of the third lumbar vertebra (L3) was used as reference point [25, 26]. The fat and muscle areas were adjusted for height (cm^2^/m^2^), which resulted in specific indices: Total fat index (TFI), visceral fat index (VFI), subcutaneous fat index (SFI) and skeletal muscle index (SMI). Visceral to subcutaneous fat ratio (VSR) was calculated by dividing visceral and subcutaneous fat area. Fat to muscle ratio (FMR) was calculated as the total fat area divided by the total muscle area. Muscle radiation attenuation (MRA) was calculated by analyzing bilateral psoas muscle density in Hounsfield units (HU) [27]. Due to the absence of universally established cut-off values, body composition parameters were categorized into sex-specific quartiles, following the approach used in previous studies [28, 29]. In addition, a standardized cut-off by Prado et al. was used for SMI [30]. Radiologic tumor response after two cycles of chemotherapy and RHT was assessed in all patients according to the response evaluation criteria in solid tumors (RECIST) 1.1 [23]. Imaging was reviewed by a radiologist with subspecialty training in oncologic imaging and extensive experience in sarcoma imaging (WGK).

### Histopathologic assessments

To evaluate the histopathologic response to preoperative treatment, resection specimens were evaluated according to standard protocols by an experienced sarcoma pathologist (TK). Parameters of interest from the resection specimen included total percentage of viable/stainable cells with a corresponding final response score as described by the EORTC Soft Tissue and Bone Sarcoma Group (EORTC-STBSG, grades A-E) [22]. 

### Statistical analysis

Survival endpoints of this study included event-free survival (EFS) and OS. The EFS duration was estimated by the time from start of chemotherapy and RHT to first progression, recurrence or death. OS was estimated by the time from start of chemotherapy to death by any cause. Cox proportional hazards regression analyses were performed to assess the association between various clinical variables and EFS/OS, with event-free patients being censored at the time of their last follow-up visit. Logistic regression analyses were conducted to evaluate the association between clinical variables and both radiologic and histopathologic treatment response. The Mann-Whitney U test was used to compare the distribution of body composition parameters between male and female patients. Pearson’s correlation coefficients were calculated to assess the relationship between different body composition parameters. A two-sided *p*-value less than 0.05 was considered statistically significant. Statistical analyses were performed using R version 4.3.3 (R Foundation for Statistical Computing, Vienna, Austria).

## Results

### Patient cohort

In total, 85 patients were analyzed (Fig. 1). The clinicopathologic characteristics of the study cohort are summarized in Table 1. Median age was 60 years, and most patients were male (56%). The most common histological subtypes were undifferentiated pleomorphic sarcoma (UPS, 45%), followed by synovial sarcoma (18%) and myxofibrosarcoma (7%). Most patients had extremity sarcomas (80%), followed by trunk (16%) and head/neck (4%) sarcomas. Most patients (62%) received preoperative radiotherapy, with a median dose of 50 Gy (Range 48–60 Gy). 94% of patients were treated with AI + RHT, while 6% of patients received AD + RHT.

**Table 1:** Demographic data

Covariate	Category	*n* (%)
**Age (years)**		Median 60 (range 24–78)
**Sex**		
	Female	37 (44)
	Male	48 (56)
**Histological subtype**		
	Undiff. Pleomorph. Sarcoma	38 (45)
	Synovial Sarcoma	15 (18)
	Myxofibrosarcoma	6 (7)
	Dediff. Liposarcoma	5 (6)
	Leiomyosarcoma	5 (6)
	Pleomorphic Liposarcoma	3 (4)
	Myxoid Liposarcoma	3 (4)
	Fibrosarcoma	3 (4)
	MPNST	3 (4)
	Other	4 (5)
**Grading**		
	G2	34 (40)
	G3	51 (60)
**Localization**		
	Extremities	68 (80)
	Trunk	13 (14)
	Head/Neck	4 (6)
**Largest diameter at baseline (cm)**		Median 7.4 (range 2.7–19.3)
**Chemotherapy protocol**		
	Doxorubicin + Ifosfamide (AI)	80 (94)
	Doxorubicin + Dacarbazine (AD)	5 (6)
**Preoperative chemotherapy cycles**		Median 4 (range 2–8)
**Preoperative radiotherapy**		
	Yes	53 (62)
	No	32 (38)
**Postoperative radiotherapy**		
	Yes	15 (18)
	No	66 (78)
	Missing	4 (5)
**Postoperative chemotherapy**		
	Yes	49 (58)
	No	36 (42)
**RECIST response after two cycles of chemotherapy + regional hyperthermia (RHT)**		
	Partial response (PR)	3 (4)
	Stable disease (SD)	68 (80)
	Progressive disease (PD)	14 (16)
**Operative resection margins**		
	R0	80 (94)
	R1	4 (5)
	Rx	1 (1)
**Histopathologic response (EORTC-STBSG)**		
	A	11 (13)
	B	4 (5)
	C	13 (15)
	D	23 (27)
	E	34 (40)

MPNST = Malignant peripheral nerve sheath tumor. RECIST = Response Evaluation Criteria In Solid Tumors. EORTC-STBSG = EORTC Soft Tissue and Bone Sarcoma Group. Other histological subtypes include 1 undifferentiated spindle cell sarcoma, 1 undifferentiated sarcoma NOS, 1 angiosarcoma, 1 epithelioid sarcoma

### Treatment response and survival

The distribution of radiologic and histopathologic treatment response can be seen in Table 1. There was no significant association between radiologic and histopathologic treatment response and survival (Supp. Table 1). Furthermore, clinical parameters such as histology, grading and preoperative radiotherapy did not have a significant impact on radiologic or histopathologic treatment response (Supp. Table 2). At a median follow-up of 50.3 months (95% CI 40.9–65.1), the median EFS and the median OS were not reached. 36 EFS events (42.4%) and 16 deaths (18.8%) were reported by the end of follow-up. In the univariate analysis, incomplete resection (R1-Rx) was the only parameter significantly associated with both worse EFS and OS (EFS: HR 4.15, 95% CI 1.60-10.78, *p* = 0.003; OS: HR 6.30, 95% CI 1.73–22.93, *p* = 0.005, supp. Table 1).

### Body composition parameters

**Fig. 2:**
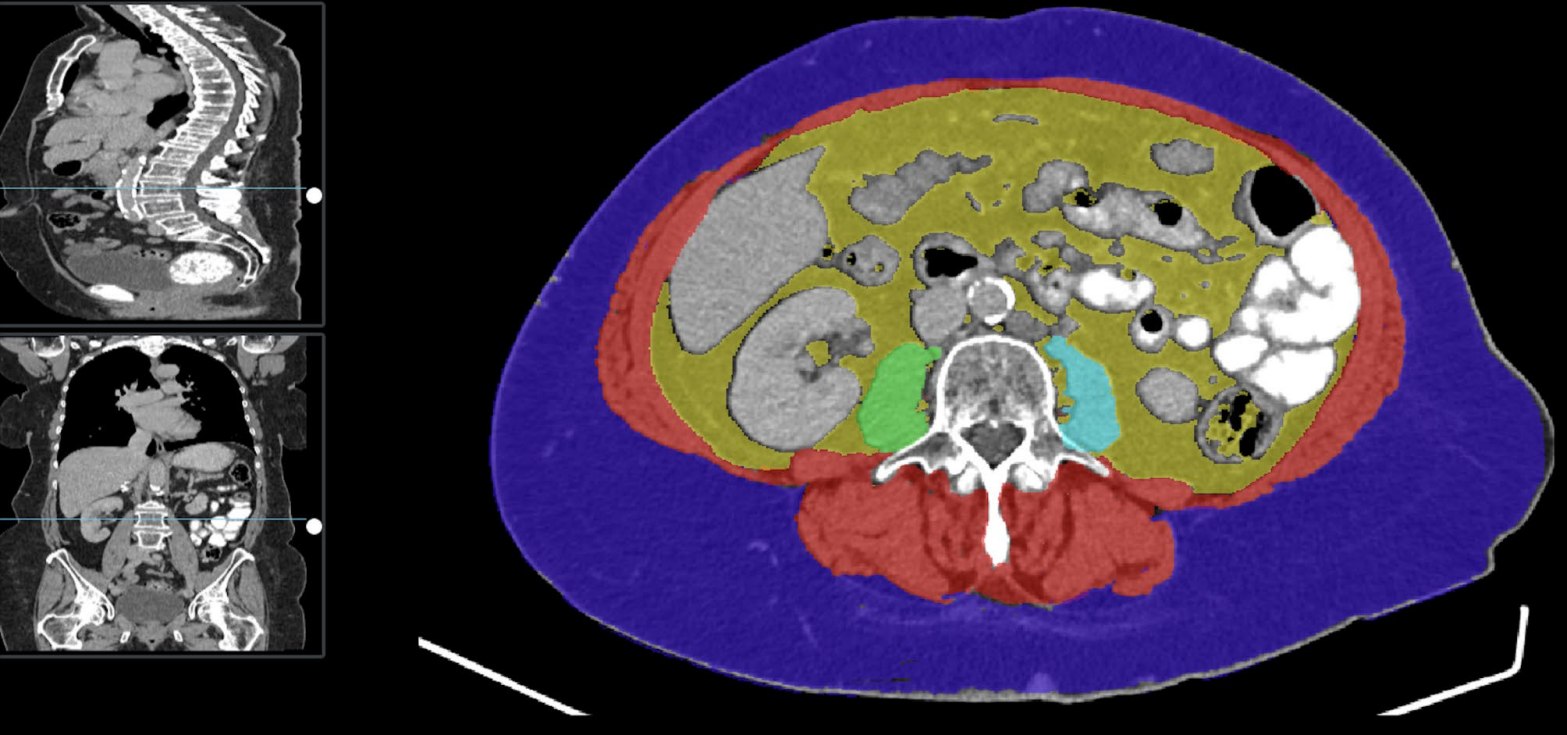
Representative example of the performed body composition analysis. The different tissue compartments were automatically colorized and analyzed

The median values of the analyzed body composition parameters can be seen in Table 2. A representative example of the performed body composition analysis can be seen in Fig. 2. According to the current World Health Organization (WHO) definition [31], 58% of patients presented as overweight or obese. A male predominance in obesity was observed (69% vs. 46% in female patients). BMI, skeletal muscle index (SMI), visceral fat index (VFI), and visceral to subcutaneous fat ratio (VSR) were significantly higher in male patients (Table 2). A higher body mass index (BMI) was associated with both higher fat and muscle indices: BMI correlated significantly with total fat index (TFI, female: *p* < 0.001, *r* = 0.87; male: *p* < 0.001, *r* = 0.86), visceral fat index and subcutaneous fat index (VFI and SFI, female: *p* < 0.001, *r* = 0.78; male: *p* < 0.001, *r* = 0.75 and female: *p* < 0.001, *r* = 0.84; male: *p* < 0.001, *r* = 0.79, respectively). BMI was significantly correlated with SMI (female patients: *r* = 0.5, *p* = 0.002, male patients: *p* < 0.001, *r* = 0.8), TFI (female: *p* = 0.047, *r* = 0.33, male: *p* < 0.001, *r* = 0.74), VFI (female: *p* = 0.022, *r* = 0.38, male: *p* < 0.001, *r* = 0.72) and SFI in men (*p* < 0.001, *r* = 0.58). The sex-specific body composition quartile cut-offs are provided as a supplementary file (Supp. Tables 3–4).

**Table 2:** Body composition parameters according to sex

Covariate	Category	Median (range)	*p* value
**Body Mass Index** (**BMI, kg/m**^**2**^)			
	Female	23.9 (19.1–44.1)	**0.039**
	Male	27.2 (18.0-38.6)	
**Muscle radiation attenuation (MRA**,** HU)**			
	Female	51.8 (31.9–67.1)	0.48
	Male	51.1 (19.6–65.4)	
**Skeletal muscle index (SMI**,** cm**^**2**^**/m**^**2**^**)**			
	Female	44.6 (30.3–64.8)	**< 0.001**
	Male	53.9 (35.2–77.9)	
**Total Fat index (TFI**,** cm**^**2**^**/m**^**2**^**)**			
	Female	112.6 (18.9-271.9)	0.74
	Male	111.0 (16.3-219.2)	
**Visceral Fat index (VFI**,** cm**^**2**^**/m**^**2**^**)**			
	Female	31.4 (1.8-112.7)	**0.0059**
	Male	60.5 (6.1-124.8)	
**Subcutaneous Fat index (SFI**,** cm**^**2**^**/m**^**2**^**)**			
	Female	71.4 (14.7-175.4)	0.080
	Male	51.0 (9.0-131.8)	
**Visceral to subcutaneous fat ratio (VSR)**			
	Female	0.4 (0.1–1.2)	**< 0.001**
	Male	1.0 (0.2–2.3)	
**Fat to muscle ratio (FMR)**			
	Female	2.4 (0.5–5.5)	0.11
	Male	2.1 (0.4–3.8)	

### Effect of body composition parameters on preoperative treatment and survival

**Fig. 3:**
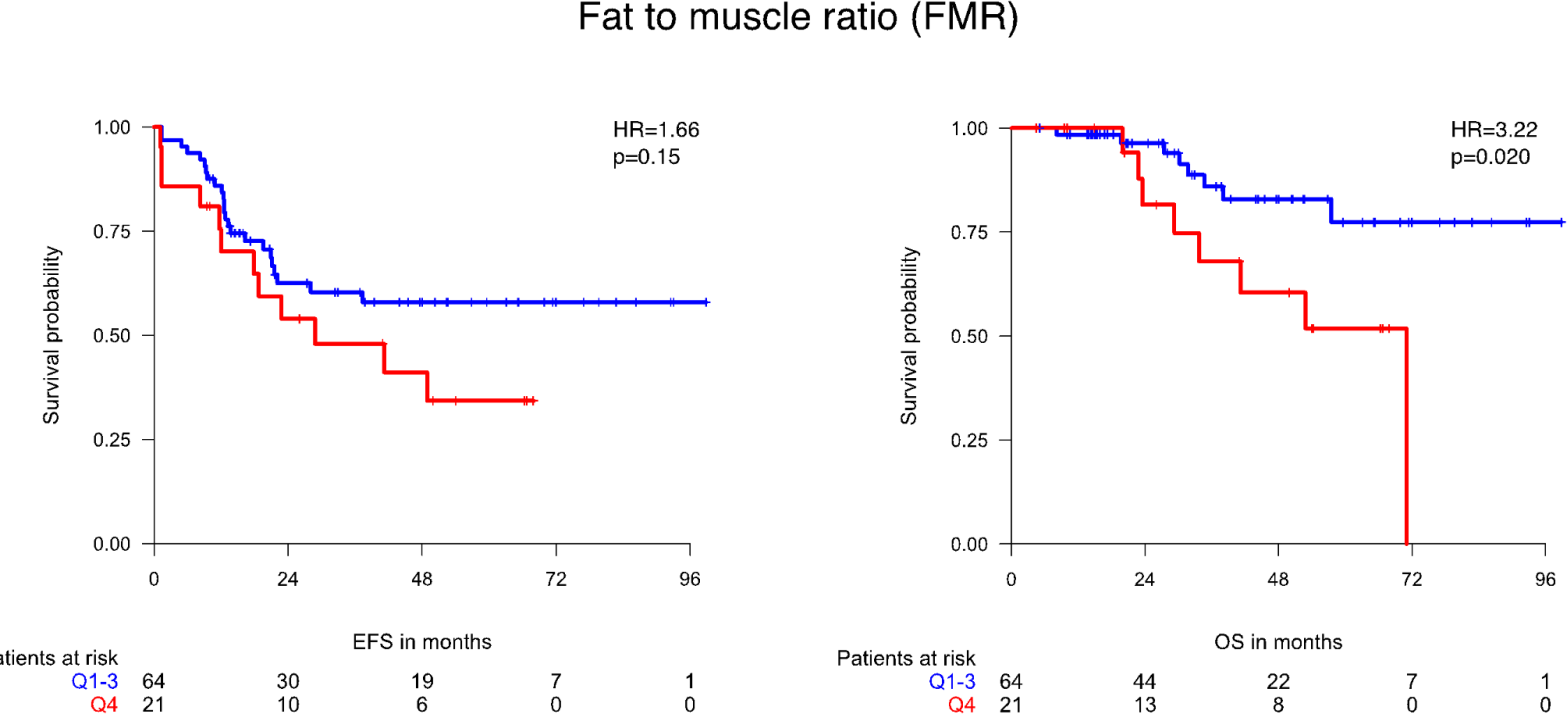
Event-free survival (EFS) and overall survival (OS) according to fat to muscle ratio (FMR)

The impact of body composition parameters on treatment response can be seen in Table 3. There was no significant association between body composition parameters and radiologic or histopathologic treatment response. High VFI showed a non-significant trend toward worse radiologic treatment response (*p* = 0.093). High TFI and SMI were significantly correlated with less chemotherapy dose reductions (*p* = 0.033 and *p* = 0.013, respectively). The effect of body composition parameters on EFS and OS can be seen in Table 4. There was no significant correlation between body composition parameters and EFS. BMI ≥ 30 (*p* = 0.017), high TFI (*p* = 0.0047), high FMR (*p* = 0.020, Fig. 3), high VFI (*p* = 0.012) and SFI (*p* = 0.012) were significantly correlated with poor OS. The association of high TFI with poor OS was confirmed in the multivariate analysis (HR 3.47, 95% CI 1.24–9.72, *p* = 0.018, Table 5).

**Table 3:** Correlation between body composition parameters and radiologic/pathologic response to treatment

Factor	Strata	Rad. Response (RECIST)		Sig.	Path. Response (EORTC-STBSG)		Sig.
		PR/SD	PD		A	B-E	
BMI (kg/m^2^)	≥ 30	14	3	0.88	1	16	0.35
	< 30	57	11		10	58	
SMI (cm^2^/m^2^)	Q1	18	4	0.80	1	21	0.20
	Q2-Q4	53	10		10	53	
SMI (cm^2^/m^2^)	Male: <52.4* Female: <38.5*	22	6	0.39	2	26	0.28
	Male: ≥52.4* Female: ≥38.5*	49	8		9	48	
TFI (cm^2^/m^2^)	Q4	16	5	0.30	3	18	0.83
	Q1-Q3	55	9		8	56	
FMR	Q4	16	5	0.30	2	19	0.59
	Q1-3	55	9		9	55	
VFI (cm^2^/m^2^)	Q4	15	6	0.093	2	19	0.59
	Q1-Q3	56	8		9	55	
SFI (cm^2^/m^2^)	Q4	17	4	0.71	2	19	0.59
	Q1-Q3	54	10		9	55	
VSR	Q4	17	4	0.71	4	17	0.34
	Q1-Q3	54	10		7	57	
MRA (HU)	Q1	19	3	0.68	3	19	0.91
	Q2-Q4	52	11		8	55	

BMI = Body mass index, SMI = Skeletal muscle index, TFI = Total fat index, FMR = Total fat to muscle ratio, VFI = Visceral fat index, SFI = Subcutaneous fat index, VSR = Visceral to subcutaneous fat ratio, MRA = Muscle radiation attenuation, HU = Hounsfield units, *=Cut-offs by Prado et al. [30]

**Table 4:** Univariate analysis of body composition parameters with regard to event-free and overall survival (EFS/OS)

Factor	Strata	EFS		OS	
		Sig.	Hazard Ratio (95%CI)	Sig.	Hazard ratio (95%CI)
BMI (kg/m^2^)	≥ 30 vs. <30	0.46	1.33 (0.62–2.82)	**0.017**	3.36 (1.25–9.08)
SMI (cm^2^/m^2^)	Q1 vs. Q2-Q4	0.34	1.43 (0.68–2.99)	0.27	0.32 (0.04–2.42)
SMI (cm^2^/m^2^)	Male: <52.4 vs. ≥52.4* Female: <38.5 vs. ≥38.5*	0.17	0.63 (0.32–1.22)	0.084	5.98 (0.79–45.39)
TFI (cm^2^/m^2^)	Q4 vs. Q1-Q3	0.16	1.64 (0.82–3.29)	**0.005**	3.56 (1.32–9.58)
FMR	Q4 vs. Q1-Q3	0.15	1.66 (0.83–3.33)	**0.020**	3.22 (1.20–8.66)
VFI (cm^2^/m^2^)	Q4 vs. Q1-Q3	0.71	1.15 (0.55–2.39)	**0.012**	2.71 (1.02–7.24)
SFI (cm^2^/m^2^)	Q4 vs. Q1-Q3	0.080	1.86 (0.93–3.72)	**0.012**	3.51 (1.31–9.39)
VSR	Q4 vs. Q1-Q3	0.11	0.49 (0.20–1.17)	0.46	0.65 (0.21–2.04)
MRA (HU)	Q1 vs. Q2-Q4	0.95	1.03 (0.49–2.13)	0.78	1.16 (0.42–3.21)

BMI = Body mass index, SMI = Skeletal muscle index, TFI = Total fat index, FMR = Fat to muscle ratio, VFI = Visceral fat index, SFI = Subcutaneous fat index, VSR = Visceral to subcutaneous fat ratio, MRA = Muscle radiation attenuation, HU = Hounsfield units, *=Cut-offs by Prado et al. [30]

**Table 5:** Multivariate analysis of EFS/OS

Factor	Strata	EFS		OS	
		Sig.	Hazard Ratio (95%CI)	Sig.	Hazard Ratio (95%CI)
Histology	Non-UPS vs. UPS	0.33	1.44 (0.70–2.96)	0.77	1.18 (0.39–3.55)
Resection margins	Incomplete vs. complete	**0.026**	3.24 (1.15–9.08)	0.077	3.68 (0.87–15.61)
TFI (cm^2^/m^2^)	Q4 vs. Q1-Q3	0.19	1.64 (0.78–3.43)	**0.018**	3.47 (1.24–9.72)
SMI (cm^2^/m^2^)	Q1 vs. Q2-Q4	0.21	1.63 (0.76–3.50)	0.46	0.46 (0.059–3.63)

TFI = Total fat index, SMI = Skeletal muscle index

## Discussion

Aim of this study was to analyze the impact of CT-based fat and muscle distribution on oncologic outcomes in patients with non-abdominal high-risk soft tissue sarcoma (HR-STS) undergoing a multimodal preoperative treatment. For the first time, the effect of CT-based body composition parameters on radiologic and histopathologic treatment response was assessed in patients with HR-STS. We were able to demonstrate sex-specific differences in body composition in our cohort. This is consistent with previous studies on fat distribution and underlines the validity of our measurements [32]. High total fat to muscle ratio (FMR) and high total fat index (TFI) were significantly associated with poor OS, while low skeletal muscle index (SMI) indicative of sarcopenia did not have a significant impact on OS. This might be due to the strong correlation between muscle and fat indices described in this cohort, which could mask a potential effect of skeletal muscle alone in this cohort. Moreover, skeletal muscle function, an important aspect of sarcopenia, was not assessed in this study.

Previous literature on obesity and oncologic outcomes has been inconclusive. While there are studies demonstrating survival benefits in obese cancer patients [33], obesity was associated with adverse outcomes and a higher incidence of postoperative complications in patients with STS [13]. One of the most common limitations of previous studies is their reliance on BMI alone, without differentiating between specific adipose tissue compartments. High fat and low muscle mass defined as a high FMR was identified as an unfavorable combination regarding oncologic outcomes in previous studies [34]. Given the strong correlation between BMI, fat, and muscle indices, the FMR may serve as a more independent prognostic factor compared to fat or muscle indices alone. Interestingly, there was no significant correlation between body composition parameters and EFS. A potential explanation could be the stronger effect of these parameters on overall health and relevant comorbidities compared to direct tumor recurrence. However, a trend towards worse EFS associated with high fat indices, particularly the subcutaneous fat index (SFI), was observed and may become more evident in a larger cohort.

Radiologic response criteria such as RECIST and pathologic response criteria based on tumor necrosis after neoadjuvant treatment such as the EORTC-STBSG scoring system have been proposed as additional predictive tools in STS, with mixed results so far [35–40]. In our study, we were not able to demonstrate a survival benefit in patients with good radiologic or histopathologic response to preoperative therapy, which supports current research on novel radiologic criteria such as the Choi classification or different tissue-based criteria such as hyalinization or fibrosis instead of tumor necrosis in STS [35, 39]. Interestingly, body composition parameters did not have a significant effect on radiologic and histopathologic response to treatment. In contrast, the correlation between treatment response and body composition, especially obesity, was observed in several solid tumors: In a study by Song et al., low BMI was an independent predictor of higher pathologic complete response (pCR) rates in colorectal cancer patients undergoing first-line chemotherapy [41]. As a potential mechanism of action, higher levels of angiogenic factors such as VEGF, IGF-1 or leptin were observed in obese patients, leading to chemoresistance and enhanced tumor proliferation [42, 43]. On the other hand, Raman et al. explained the decrease in pCR rates in obese breast cancer patients with a higher rate of chemotherapy dose reductions due to more frequent chemotherapy-associated adverse events [44]. In our cohort, patients with high fat indices were less likely to receive dose reductions. A potential reason for the missing correlation between body composition parameters and histopathologic response to treatment in our study could be the rather small cohort and mix of different histological subtypes. A more relevant explanation could be the incomplete understanding of pathologic response to treatment in STS. We do currently not fully understand the effect of chemo- and radiotherapy on the extent of tumor necrosis, which limits the interpretation of our results. The same limitation applies to the association of radiologic response to preoperative treatment and body composition, as there was no significant correlation between the RECIST criteria and obesity or sarcopenia in our cohort. In contrast to chemo- and radiotherapy, several studies have demonstrated an association between response to immune checkpoint inhibitors and body composition parameters such as a high visceral fat index (VFI) in solid cancers, which is thought to be related to the immunogenic environment in the visceral fat compartment [21, 45]. This does not seem to apply to STS patients undergoing conventional chemotherapy and RHT.

In addition to the classical limitations of retrospective data analysis, the inclusion of various histological subtypes limits the interpretation of radiologic and histopathologic response to preoperative treatment. Moreover, due to the lack of standardized cut-off values for fat and muscle indices, we chose to use sex-specific quartiles, which complicates the reproducibility of our findings. However, when comparing our established CT-based criteria for sarcopenia to the ones used in previous studies, we found only small differences in cut-offs [12]. Furthermore, comorbidities and performance status were not reported in a standardized form. However, perioperative chemotherapy combined with RHT is typically applied to fit patients. Strengths of our study include the large and well-characterized cohort of patients with non-abdominal HR-STS. We only included patients with extremity, trunk or head/neck tumors and excluded retroperitoneal sarcoma to avoid measurement errors due to the analysis of CT scan images on the L3 level. The use of an AI-based software (CoreSlicer, version 1.0, Montreal, Canada) resulted in a valid and reproducible approach in the analysis of several body composition parameters.

## Conclusion

We provide an in-depth analysis of the impact of CT-based body composition parameters on preoperative treatment response and survival in patients with HR-STS. High fat indices and a high fat to muscle ratio (FMR) were strong predictors of poor OS, whereas radiologic and histopathologic treatment responses were not significantly influenced by body composition. Larger studies are warranted to further clarify the prognostic and predictive value of these findings.

## Electronic supplementary material

Below is the link to the electronic supplementary material.

Supplementary Material 1

## Data Availability

The data presented in this study are available on specific request from the corresponding author. The data are not publicly available for reasons of data protection and data economy.
