# Supplementary material for: The impact of CT-based adipose tissue distribution and sarcopenia on treatment outcomes in patients with high-risk soft tissue sarcoma
Source: BMC Cancer. 2025 Apr 11;25:671. doi: 10.1186/s12885-025-14050-x (PMC11992814; doi:10.1186/s12885-025-14050-x)
Supplement: Supplementary file 1 — Supplementary Material 1 [file 12885_2025_14050_MOESM1_ESM.docx]

**Supplementary file**

Table 1: Univariate analysis of clinical parameters on event-free and overall survival (EFS/OS)

|  |  | **EFS** | | **OS** | |
| --- | --- | --- | --- | --- | --- |
| **Factor** | **Strata** | **Sig.** | **Hazard Ratio (95%CI)** | **Sig.** | **Hazard ratio**  **(95%CI)** |
| Age | ≤60 vs. >60 | 0.66 | 0.86 (0.45-1.66) | 0.12 | 0.43 (0.15-1.23) |
| Sex | Male vs. Female | 0.80 | 1.09 (0.56-2.11) | 0.60 | 0.77 (0.29-2.06) |
| Histology | Non-UPS vs. UPS | 0.13 | 1.70 (0.86-3.37) | 0.40 | 1.54 (0.57-4.18) |
| Grading | G3 vs. G2 | 0.58 | 1.21 (0.62-2.37) | 0.47 | 1.47 (0.51-4.25) |
| Tumor site | Trunk/Head Neck vs. Extremity | 0.29 | 0.60 (0.23-1.54) | 0.31 | 0.35 (0.04-2.68) |
| Resection margins | R1-Rx vs. R0 | **0.003** | 4.15 (1.60-10.78) | **0.005** | 6.30 (1.73-22.93) |
| Preop. Radiotherapy | Yes vs. no | 0.86 | 0.94 (0.48-1.83) | 0.75 | 1.18 (0.44-3.18) |
| Rad. Response (RECIST) | PD vs. PR/SD | 0.42 | 1.41 (0.62-3.21) | 0.78 | 1.19 (0.34-4.21) |
| Path. Response (EORTC) | B-E vs. A | 0.80 | 1.15 (0.40-3.24) | 0.39 | 2.43 (0.32-18.52) |

Table 2: Correlation between clinical parameters and radiologic (RECIST) or pathologic (EORTC-STBSG) response to treatment

|  |  | **Rad. Response**  **(RECIST)** | | **Sig.** | **Path. Response**  **(EORTC-STBSG)** | | **Sig.** |
| --- | --- | --- | --- | --- | --- | --- | --- |
| **Factor** | **Strata** | **PR/SD** | **PD** |  | **A** | **B-E** |  |
| Age | ≤60 | 34 | 7 | 0.89 | 8 | 36 | 0.15 |
|  | >60 | 37 | 7 |  | 3 | 38 |  |
| Sex | Female | 34 | 3 | 0.080 | 5 | 32 | 0.89 |
|  | Male | 37 | 11 |  | 6 | 42 |  |
| Histology | UPS | 29 | 9 | 0.11 | 8 | 30 | 0.057 |
|  | Non-UPS | 42 | 5 |  | 3 | 44 |  |
| Grading | G2 | 31 | 3 | 0.13 | 1 | 33 | 0.052 |
|  | G3 | 40 | 11 |  | 10 | 41 |  |
| Tumor site | Trunk/Head and Neck | 15 | 2 | 0.56 | 2 | 15 | 0.87 |
|  | Extremity | 56 | 12 |  | 9 | 59 |  |
| Preop. Radiotherapy | Yes | 46 | 7 | 0.30 | 8 | 45 | 0.45 |
|  | No | 25 | 7 |  | 3 | 29 |  |

Table 3: Male quartile values of body composition parameters

|  | **Quartiles (male)** | | | |
| --- | --- | --- | --- | --- |
| **Factor** | **Q1** | **Q2** | **Q3** | **Q4** |
| SMI  (cm^2^/m^2^) | [35.19,49.36[ | [49.36,53.92[ | [53.92,63.91[ | [63.91,77.89] |
| TFI  (cm^2^/m^2^) | [16.27-72.08[ | [72.08-110.97[ | [110.97-159.36[ | [159.36-219.16] |
| FMR | [0.44-1.55[ | [1.55-2.12[ | [2.12-2.58[ | [2.58-3.76] |
| VFI  (cm^2^/m^2^) | [6.05-29.77[ | [29.77-60.53[ | [60.53-76.15[ | [76.15-124.84] |
| SFI  (cm^2^/m^2^) | [8.95-36.15[ | [36.15-51.04[ | [51.04-78.15[ | [78.15-131.79] |
| VSR | [0.23-0.66[ | [0.66-0.97[ | [0.97-1.26[ | [1.26-2.33] |
| MRA  (HU) | [19.63-42.74[ | [42.74-51.08[ | [51.08-55.26[ | [55.26-65.41] |

Table 4: Female quartile values of body composition parameters

|  | **Quartiles (female)** | | | |
| --- | --- | --- | --- | --- |
| **Factor** | **Q1** | **Q2** | **Q3** | **Q4** |
| SMI  (cm^2^/m^2^) | [30.28-39.92[ | [39.92-44.62[ | [44.62-49.33[ | [49.33-64.76] |
| TFI  (cm^2^/m^2^) | [18.91-57.24[ | [57.24-112.59[ | [112.59-160.78[ | [160.78-271.90] |
| FMR | [0.45-1.58[ | [1.58-2.44[ | [2.44-3.72[ | [3.72-5.54] |
| VFI  (cm^2^/m^2^) | [1.77-14.01[ | [14.01-31.44[ | [31.44-56.91[ | [56.91-112.69] |
| SFI  (cm^2^/m^2^) | [14.72-46.60[ | [46.60-71.36[ | [71.36-106.37[ | [106.37-175.41] |
| VSR | [0.10-0.34[ | [0.34-0.40[ | [0.40-0.64[ | [0.64-1.20] |
| MRA  (HU) | [31.87-42.66[ | [42.66-51.80[ | [51.80-57.69[ | [57.69-67.08] |
